# Single crystalline cylindrical nanowires – toward dense 3D arrays of magnetic vortices

**DOI:** 10.1038/srep23844

**Published:** 2016-03-31

**Authors:** Yurii P. Ivanov, Andrey Chuvilin, Laura G. Vivas, Jurgen Kosel, Oksana Chubykalo-Fesenko, Manuel Vázquez

**Affiliations:** 1 King Abdullah University of Science and Technology (KAUST), Thuwal, 23955, Saudi Arabia; 2 Instituto de Ciencia de Materiales de Madrid, CSIC, Cantoblanco, 28049, Madrid, Spain; 3 CIC nanoGUNE Consolider, Av. de Tolosa 76, 20018, San Sebastian, Spain; 4 IKERBASQUE, Basque Foundation for Science, Maria Diaz de Haro 3, 48011, Bilbao, Spain; 5 Physics and Materials Science Research Unit, University of Luxembourg, 162A Avenue de la Faïencerie, L-1511, Luxembourg

## Abstract

Magnetic vortex-based media have recently been proposed for several applications of nanotechnology; however, because lithography is typically used for their preparation, their low-cost, large-scale fabrication is a challenge. One solution may be to use arrays of densely packed cobalt nanowires that have been efficiently fabricated by electrodeposition. In this work, we present this type of nanoscale magnetic structures that can hold multiple stable magnetic vortex domains at remanence with different chiralities. The stable vortex state is observed in arrays of monocrystalline cobalt nanowires with diameters as small as 45 nm and lengths longer than 200 nm with vanishing magnetic cross talk between closely packed neighboring wires in the array. Lorentz microscopy, electron holography and magnetic force microscopy, supported by micromagnetic simulations, show that the structure of the vortex state can be adjusted by varying the aspect ratio of the nanowires. The data we present here introduce a route toward the concept of 3-dimensional vortex-based magnetic memories.

Magnetic vortices are objects of rotational symmetry composed of a relatively small core (typically < 10 nm), where the magnetization points out of the sample plane, surrounded by in-plane (e.g., circumferential) magnetization^1^,^2^. The vortex state occurs in thin ferromagnetic square and disk-shaped elements (“dots”) that are micrometer-sized and smaller. The stability of the vortex in the disk depends substantially on the exchange length of the material and the aspect ratio (diameter/thickness)^3^. The vortex state is very stable against thermal fluctuations because of the height of the energy barrier that separates the two states with opposite polarities and because it involves the Bloch point^4^. Meanwhile, the polarization of the vortex core can easily be controlled by its resonant excitation with small bursts of an alternating^5^ or in-plane rotating GHz magnetic field^6^ or by spin-polarized currents^7^. These properties suggest that magnetic dots could be appropriate as building blocks for new spintronic devices. The first device proposed was a memory cell, where data bits can be stored in spin directions in the nanometer-scale-sized core^8^,^9^,^10^,^11^. More recently, an application related to vortex-based nanooscillators working in the sub-gigahertz regime was suggested^12^,^13^. The practical realization of these applications will depend on the ability to create low-cost, large-scale media that have a high density of magnetic elements with dimensions down to a few 10ths of a nm and that have a stable vortex state. Such planar structures can be integrated into potential new spintronic devices using well-established electron beam lithography; however, this restricts structures to the existing 2-dimensional device paradigm. By developing 3-dimensional (3D) media^14^,^15^ that can maintain vortex states localized to a few 10ths of a nm, a new technological concept for high-density spintronic devices will be born. In 2014, we used simulations to propose^16^ that specifically oriented Co nanowires (NWs) in a crystal structure may maintain a vortex state along their entire length, making them an excellent candidate for vortex-based media. Shorter nanopillars could be prepared by either controlled deposition conditions^17^ or simply by etching the NW array.

Cylindrical NWs with diameters between 15–200 nm and high aspect ratios can be prepared by electrodeposition into templates. For instance, hexagonally ordered arrays of NWs with high packing density can be fabricated using anodic aluminum oxide (AAO) membranes^17^,^18^,^19^. Typically, polycrystalline, magnetically soft NWs (such as a permalloy, *fcc* Co, Fe, or Ni) have shape anisotropy and remain at remanence in a near-single domain state when a field is applied parallel to their axes with an “open” vortex-like structure at their ends^16^,^20^. Although only recently studied experimentally, starting with these open vortex structures, a magnetization reversal occurs via propagation of a Bloch-point vortex domain wall (VDW)^21^, similar to micromagnetic modeling, which was proposed ten years earlier^20^. Thus, although vortices in NWs is not a novel concept, they are related with dynamic processes. To stabilize these VDWs, NWs can be pinned to the location of a defect, making them difficult to control. Our group only recently predicted the existence of stable magnetic vortices at remanence along the full length of cylindrical, single-crystal *hcp* Co NWs using micromagnetic simulations^22^; to date, no direct experimental evidence of their existence at remanence has been reported.

Here, we report the first direct experimental proof of the existence of stable magnetic vortex states at remanence in cylindrical, single-crystal *hcp* Co NWs by Lorentz microscopy (LorTEM) and electron holography. Using this system, we are able to present a 3D concept of magnetic vortex media for multiple applications in nanotechnology.

## Results and Discussion

### Structural study

Self-assembled nanopores with long-range, hexagonal packing were grown by a controlled two-step anodization process of highly pure aluminum disks in oxalic acid^23^. Pore size varied between 45 and 75 nm using an appropriate chemical etching time in phosphoric acid. The average pore center-to-center distance was measured at ~105 nm.

Pores were filled with Co by controlled electrodeposition in a suitable bath (see Supplementary Information). NWs were approximately 10-μm long. Supplementary Fig. S1a shows a typical cross-sectional image of a Co NW array embedded into an AAO membrane. For all samples, X-ray diffraction (XRD) with Cu K_α1_ radiation (λ = 1.54056 Å) identified *hcp* crystal phases with a pronounced peak at 41.63° and a small peak at 75.96°, corresponding to (100) and (2–10) planes of *hcp* Co, respectively (Supplementary Fig. S1b). The absence of {00l}-type reflections indicates that the c-axis is oriented perpendicular to the NW axis^24^. Analyses of scanning electron microscopy (SEM) and transmission electron microscopy (TEM) images showed NWs with a straight, uniform diameter, a smooth surface, and a single-crystal structure along their full length. This was confirmed by convergent beam electron diffraction patterns that were acquired sequentially along the length of randomly selected NWs (see Supplementary Fig. S3).

**Figure 1:**
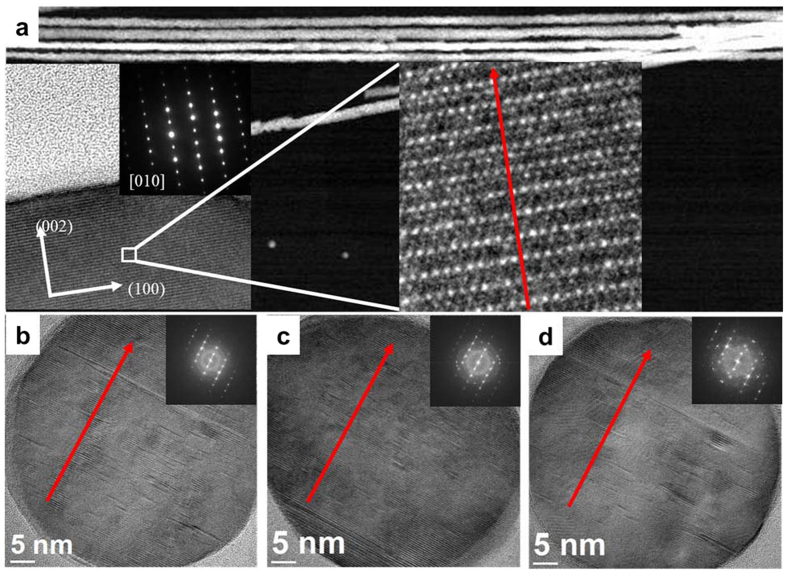
(**a**) Scanning TEM image of 75-nm diameter single-crystal Co NWs. In the insert the HRTEM and corresponding SAED images are shown. (**b–d**) HRTEM images in the plane perpendicular to the NW axis for several nanowires inside the membrane. Red arrow show the orientation of the c-axis.

The *hcp* Co phase is characterized by strong uniaxial magnetocrystalline anisotropy in the direction of the c-axis. As shown by both the high-resolution TEM (HRTEM) and the selected-area electron diffraction (SAED) pattern in Fig. 1, the c-axis is almost perpendicular to the NW axis, in agreement with results from the XRD. Hence, as predicted by the magnetic state diagram in ref. 22 a strong competition between shape and magnetocrystalline anisotropies is expected and a magnetic vortex state should exist at remanence. Here, we should note that a comparative analysis of the hysteresis loops in Supplementary Fig. S1c, measured by vibrating sample magnetometry under parallel (||) and perpendicular (⊥) fields to the NW axis, showed no clear easy magnetization axis direction. In addition, remanence of arrays of NWs with a 45-nm diameter after saturation by the field parallel to the NW axis was only slightly higher than that for arrays of NWs with a 75-nm diameter.

### Lorentz microscopy and electron holography study

The magnetic state of individual magnetic NWs can be assessed using magnetic force microscopy (MFM)^24^,^25^ or X-ray magnetic circular dichroism^21^. For NWs with diameters less than 100 nm, LorTEM and/or electron holography are an alternative suitable approach^26^. The Fresnel mode of LorTEM is achieved by observing the sample in a plane far from the ordinary image plane. Depending on their chirality, magnetic vortices act as convex or concave lenses, having either a real focus below or virtual focus above the sample. In the LorTEM image, this is visualized as bright spots in the center of the vortex either in over-focused or under-focused conditions^27^. The amount of defocusing should be selected with consideration for the intensity of the magnetic induction, specimen thickness, and coherency of electron waves. Electron holography, in turn, directly measures the phase shift of the electron wave passing through the sample^28^,^29^ by measuring the phase shifts on the interferogram. This technique can be used to obtain quantitative information about magnetic and electric fields in materials with nm-scale spatial resolution^30^. Both methods are sensitive only to in-plane (perpendicular to electron beam) components of the magnetic field.

**Figure 2:**
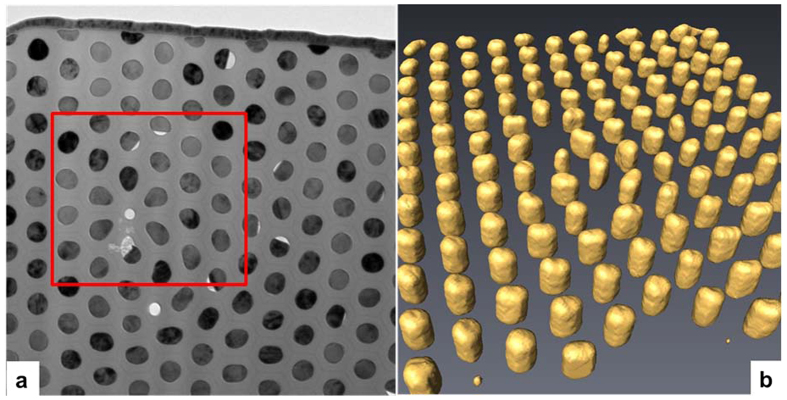
(**a**) BF-TEM and (**b**) reconstructed tomography image of an array of single-crystal *hcp* Co NWs with a 45-nm diameter and a 55-nm length.

We prepared parallel sections of the AAO membranes with embedded NWs using a focused ion beam (FIB) system to observe the magnetic structures by LorTEM and electron holography (see Supplementary Information). The sectional sample was prepared as a wedge with the thickness gradually increasing from 20 to 200 nm. Increasing thickness along the wedge was measured using the images recorded at a sample tilt angle of 30°. Consequently, we investigated segments of NWs from 20 to 200 nm in length. At selected positions, a ±75° tomography series was acquired and reconstructed for a better estimation of NW shape. Figure 2 shows the bright-field TEM (BF-TEM) image (a) and a reconstructed tomography image (b) of the cross section of the array of Co NWs with a 45-nm diameter and an estimated length of 55 nm.

Although most NWs were nearly cylindrical in shape, such that they made circular cross-sections (see Figs 1b–d and 2b), we also observed some with an imperfectly circular shape, seemingly due to the presence of defects in AAO membranes formed during anodisation of the aluminum disk. We used one of these defects to identify an area of interest for correlating data obtained by different methods.

**Figure 3:**
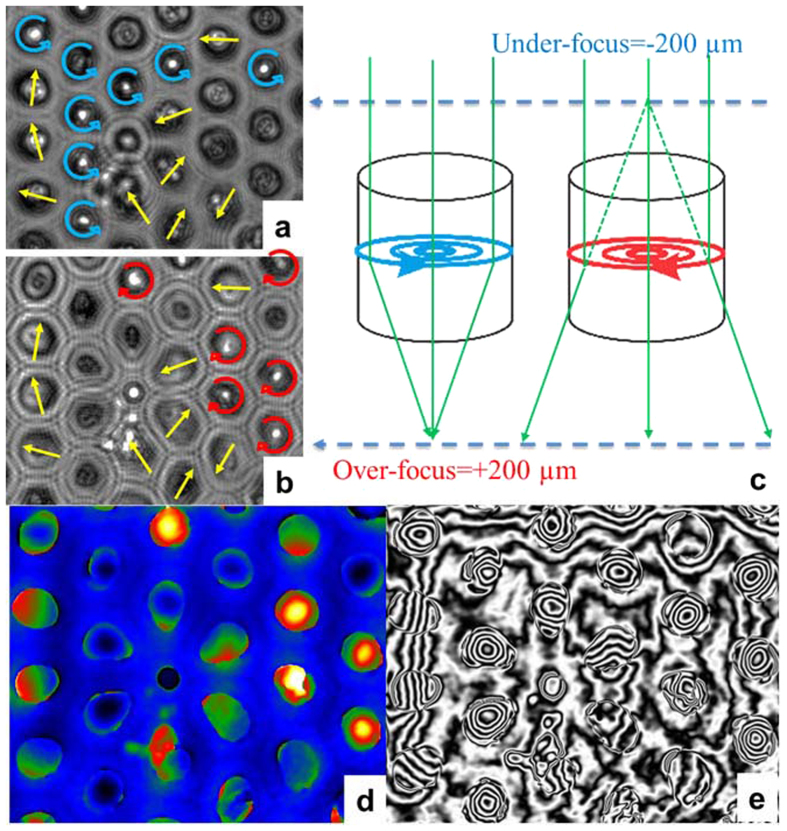
LorTEM images in over- (**a**) and under-focused (**b**) conditions of an array of NWs 45 nm in diameter and 55-nm long at remanence. The arrows show the transverse direction and the clockwise (red) and anticlockwise (blue) rotation of NW magnetization. As (**c**) shows schematically, the magnetic vortex acts as a convex or a concave lens, depending on its chirality, which creates a focus above or below the sample. Looking at these planes, we could detect the presence of a vortex state and determine its chirality. On the hologram image (**d**) the high- and low-phase values are represented by two color sequences that correspond to the clockwise and anticlockwise rotation of NW magnetization and (**e**) shows the contour lines corresponding to the B⊥.

LorTEM images corresponding to the square region (marked in red in Fig. 2a) of the array of single-crystal *hcp* Co NWs are shown in Fig. 3a,b. We observed that most of the NWs with a circular cross section had a bright spot in the center either in over- or under-focused conditions, indicating the presence of vortices with different chirality. Some NWs had broader spots (Fresnel fringes due to differences in electrostatic potential in the Co and alumina), whose positions shifted slightly in over- and under-focused conditions. These shifts are indicative of the presence of a linear, in-plane magnetic-field component that is perpendicular to the shift vector. Thus, we concluded that some NWs were partly magnetized in plane of the section while others contained vortices of different chirality.

We confirmed these observations by holographic measurements. The phase gradient measured from the interferogram was directly proportional to the perpendicular component of the magnetic induction vector (B⊥) in the specimen, and a graphic representation of the strength and direction of the locally projected B⊥ can be obtained by simply adding contours to the recorded magnetic contribution of the phase image. The field lines in Fig. 3e, obtained from the hologram, reveal circular contours, which correspond to in-plane circularly magnetized vortices. Some of the NWs show a magnetic state with a strong in-plane magnetization component, while a few are either nonmagnetic or have no in-plane component. Two color sequences in Fig. 3d were used to highlight the clockwise (red to yellow) and anticlockwise (green to blue) rotation of NW magnetization, which is also schematically shown in Fig. 3a,b. Yellow arrows indicate the direction of the in-plane component for NWs without a vortex state.

Consequently, the remanent state of NWs with a 45-nm diameter and an aspect ratio (length/diameter) from 0.5 (20-nm long NWs) to 5 (200-nm long NWs) is either a vortex or a single-domain magnetization perpendicular to the NW axis. In comparison, the ground state of lithographically fabricated permalloy, disk-shaped elements with such dimensions is a single-domain magnetized in parallel with the NW axis^3^.

**Figure 4:**
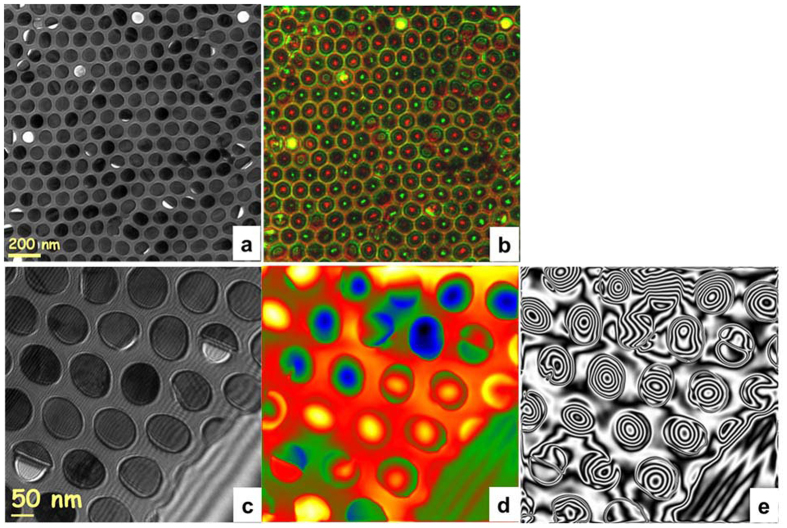
(**a**) BF-TEM and (**b**) mixed LorTEM images at over- (+200 μm, red spots) and under-focused (−200 μm, green sports) conditions of an array of NWs with a 75-nm diameter at remanence (red and green spots correspond to NWs with opposite magnetization chirality). (**c**) The original interferogram, (**d**) the reconstructed image of an array of NWs with a 75-nm diameter at remanence (high- and low-phase values are represented by two color sequences corresponding to the clockwise and anticlockwise rotation of NW magnetization) and (**e**) shows the contour lines that correspond to the B⊥.

To stabilize the vortex state, we increased the diameter of NWs to 75 nm, as shown in Fig. 4. Figure 4b shows the mixed over- and under-focused images of the NW array presented in Fig. 4a (red and green spots correspond to NWs with opposite magnetization chirality). Only a few NWs exhibited a ground state different from the vortex state, and as shown in the reconstructed hologram image in Fig. 4e, which is a magnetization in plane with NW diameter. Consequently, the number of NWs with a vortex state was much larger than that of those with an in-plane state.

The contours in Figs 3e and 4c provide a quantitative measure of the strength and direction of the local magnetic field. Two contour lines contain a particular magnetic flux: with a known distance between lines and sample thickness, the magnetic field at each point can be calculated. Taking into account the thickness of the NW cross-sections measured from the tomogram, the in-plane component of the magnetic induction calculated from the hologram in Figs 3e and 4c amounts to 1.28 T and 1.75 T for NWs with a vortex state and 45-nm and 75-nm diameters, respectively (see Supplementary Information). Considering a saturation magnetization of bulk *hcp* Co of 1.76 T, we can conclude that the magnetization lies completely in plane (i.e., in the vortex for NWs with a 75-nm diameter). However, the magnetization of the vortex shell for NWs with a 45-nm diameter contains a component parallel to the NW axis. Note that the very small value of the in-plane magnetic induction between 45-nm NWs in the array suggests a nearly negligible dipole-dipole interaction in the array; 75-nm NWs also had very small stray fields when in a vortex state but had comparatively strong stray fields with a strong in-plane magnetization component.

### Micromagnetic simulations

**Figure 5:**
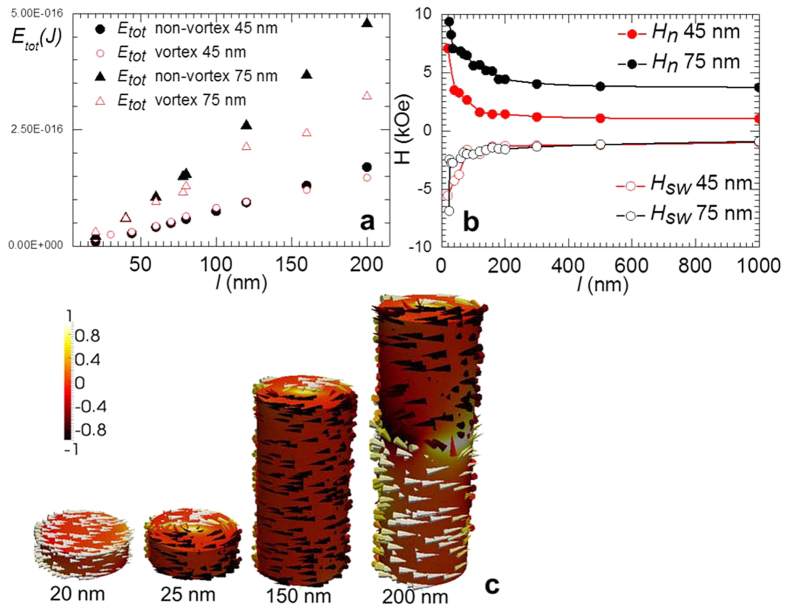
(**a**) Calculated dependence of the total energy of the vortex and non-vortex state on the length of NWs. (**b**) The nucleation field of the vortex and the switching field for the vortex core as a function of NW length after saturation in 1 T parallel to the NW axis. (**c**) Simulated magnetization of single-crystal hcp Co NW with a 75-nm diameter, depending on the length of the NW.

We performed micromagnetic simulations with the typical magnetic parameters of bulk *hcp* Co (saturation magnetization M_s_ = 1.76 T, exchange stiffness constant A = 1.3 × 10^−11^ J/m and uniaxial magnetic anisotropy value K = 4.5 × 10^5^ J/m^3^) to understand the occurrence of different magnetic states and the magnetization process^22^. Following TEM measurements we have assumed that the nanowires are single crystalline with c-axis oriented at 88° with respect to the nanowire length. After saturation parallel to the NW axis by a 1 T magnetic field, the magnetization reversal started by the nucleation of the vortex at a still positive magnetic field. At a certain negative field, the vortex core irreversibly switches, and for the rest of the process, the spins in the vortex shell have a reversible rotation towards the negative magnetic field. Figure 5b shows the calculated nucleation field (H_n_) and switching field (H_sw_) versus NW length. H_n_ and H_sw_ drastically decrease as aspect ratio (l/d) increases up to 2, at which point their values saturate. A higher value of shape anisotropy, capable of competing with magnetocrystalline anisotropy for NWs with a 45-nm diameter, results in significantly smaller values of H_n_ compared with those for NWs with a 75-nm diameter, but practically does not show up in the H_sw_ dependence. Thus, the values of the critical fields of the vortex can easily be tuned by adjusting the aspect ratio of NWs. In Fig. 5c we present the minimum energy configurations for different NW aspect ratios.

**Figure 6:**
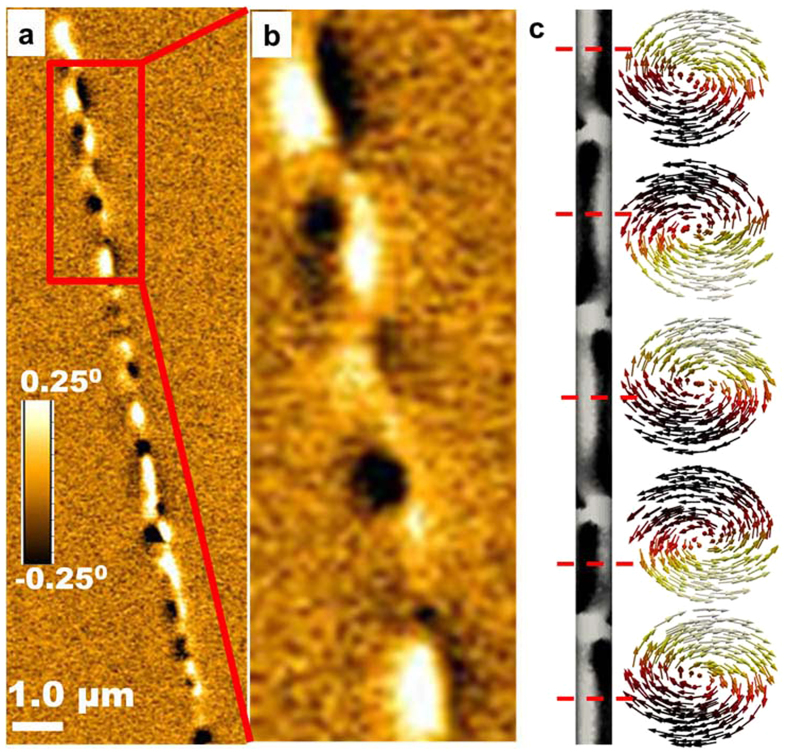
(**a**) A MFM image of a NW with a 45-nm diameter and a 10-μm length at remanence. (**b**) A close up of an area with five alternating vortices and (**c**) the corresponding results of a micromagnetic simulation. (The colors along the nanowire correspond to the MFM contrast obtained and the arrows show the clockwise and anticlockwise rotation of NW magnetization in adjacent vortices).

Furthermore, simulations indicated that two stable magnetic states—the vortex state and the magnetic state—with in-plane magnetization (perpendicular to the wire axis) coexist and have similar energies (see Fig. 5a). The calculations show that for NWs with a 45-nm diameter, the vortex state had the lowest energy for 100-nm NWs or longer, while for NWs with a 75-nm diameter the vortex state was most favorable at 50 nm in length. In a non-vortex state with in-plane magnetization, both diameters of NWs were favorable with lengths below 20-nm. For intermediate lengths (i.e., above the critical aspect ratio of 0.7 and 2.2 for NWs with 75-nm and 45-nm diameters, respectively), the energy associated with a vortex structure was very similar to that needed to align the magnetization mostly in plane with the NW diameter. Thus, for a range of aspect ratios close to those values, we can expect the occurrence of one or another state in different NWs in the array to agree well with the experimental data in Figs 3 and 4. Simulations show that for aspect ratios >2 the ground state may also include NWs separated along their length in two domains with two vortices with the same polarity and opposite chirality. For sufficiently long lengths, multiple vortices with alternative chirality will minimize the energy. Figure 6a,b show the MFM image of NWs with a 45-nm diameter and a 10-μm length at remanence. The alternating contrast corresponds to a magnetic state consisting of vortices with alternating chirality, as confirmed by results from the micromagnetic simulation shown in Fig. 6c. Due to the presence of a strong magneto-crystalline anisotropy, these vortices are not axially symmetrical; therefore, they produce a stray field that is observable by MFM.

Furthermore, in agreement with electron holography studies, simulations show that the magnetization component parallel to NW length is practically zero for NWs with a 75-nm diameter, while those with a 45-nm diameter varied from 0.15 to 0.35 with increasing length (from 20 to 200 nm). This can be understood considering the increasing shape anisotropy for smaller diameter or longer NWs that eventually dominate the magnetocrystalline anisotropy. This also indicates that the vortex shell is not in plane according to typical planar dot geometry.

**Figure 7:**
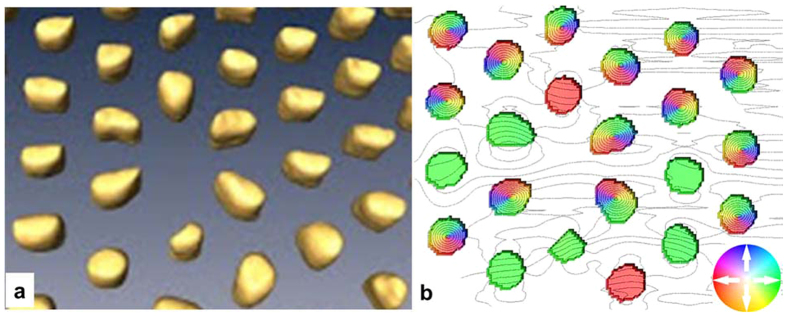
(**a**) A tomographic image of the section of the NW array presented in Fig. 3 and (**b**) the calculated ground state of magnetization (colors correspond to the in-plane component of magnetization and lines correspond to the B⊥).

We used tomographic data (see Fig. 7a) to run micromagnetic simulations on the part of the array presented in Fig. 3 to account for the magnetostatic interaction between NWs in the array and to define the true shape of NWs. As shown in Fig. 7b, NWs can present either vortex or parallel magnetization states, which agrees well with experimental data (Fig. 3).

**Figure 8:**
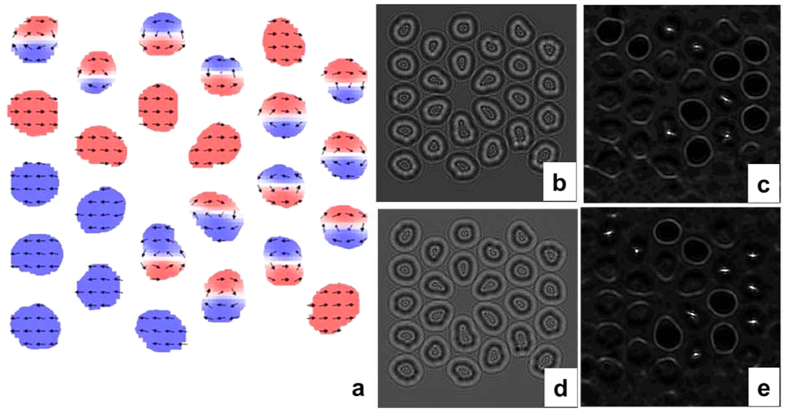
(**a**) The ground magnetic state on the NW array presented in Fig. 3 (color corresponds to the magnetization component in plane with NW diameter). LorTEM images of (**b,d**) calculated electrostatic and (**c,e**) magnetic contributions. (**b,c**) show under- and (**d,e**) show over-focused images.

**Figure 9:**
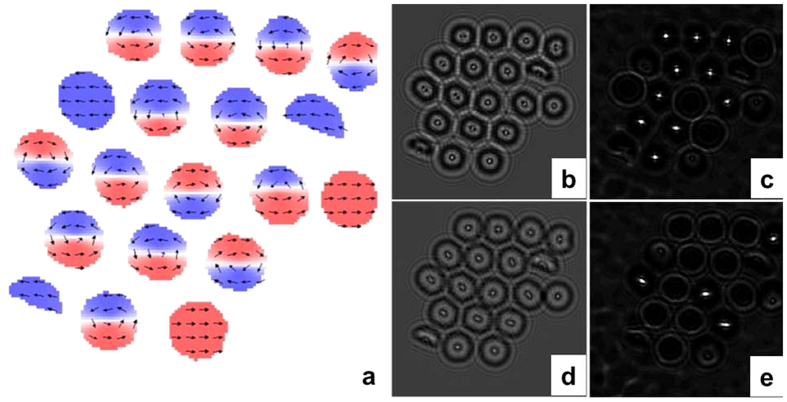
(**a**) The ground magnetic state on the NW array presented in the Fig. 4 (color corresponds to the magnetization component in plane with NW diameter). LorTEM images of (**b,d**) calculated electrostatic and (**c,e**) magnetic contributions. (**b,c**) show under- and (**d,e**) show over-focused images.

The spin configuration extracted from micromagnetic simulations was also used to simulate the LorTEM images for comparison with the experimental images shown in Figs 3a,b and 4a,b. This simulation clearly showed that NWs with vortices behave like a lens, focusing the electron beam (Figs 8a,c,e and 9a,c,e). Any other contrast in the LorTEM images can be attributed to inner electrostatic potential (Figs 8b,d and 9b,d).

## Conclusion

Arrays of hexagonally ordered, magnetic vortices were created in monocrystalline *hcp* Co NWs grown using a simple electrochemical technique. This type of vortex structure is achieved by a competition between shape and crystalline anisotropies. We used holography and LorTEM studies to observe the results.

The vortex state is observed in arrays of NWs with diameters as small as 45 nm and lengths of 200 nm, for which cross talk between neighboring vortices was observed to disappear. This is an important difference compared to permalloy dots and nanopillars, which show no vortex state at similar dimensions.

Simulations show that a stable vortex state is obtained for NWs with dimensions that exceed a critical aspect ratio. They also show that multiple vortices with different chirality can exist along NWs with higher aspect ratios. This intriguing quality for data storage media was confirmed by MFM measurements.

The simplicity and efficiency of the vortex structures fabricated in this study are motivation for the continued exploration into new opportunities for the use of an advanced 3D magnetic vortex memory system.

## Methods

### Growth of Co nanowires

AAO membranes with highly ordered, hexagonal, self-assembled nanopore arrays were prepared by a two-step anodization process in oxalic acid^23^. Prior to anodization, high-purity (99.999%) aluminum disks were degreased in acetone by ultrasound and cleaned by electropolishing in a mixture of perchloric acid and ethanol (HClO_4_:C_2_H_5_OH = 1:4 in volumetric ration) for 2 min at 6 °C with vigorous stirring. Afterwards, samples were rinsed in an ethanol solution and dried. The first anodization procedure was performed using a 0.3-M oxalic acid solution as an electrolyte at 3 °C and an anodization voltage of 40 V. After the first anodization, the sample was immersed in a chromic acid/phosphoric acid mixture at room temperature until the oxide layer was dissolved. The second anodization was performed for 20 h resulting in pore depths of up to 40 μm. Next, time-controlled treatments in phosphoric acid increased pore diameters to 45 and 75 nm. Then, the non-oxidized Al and alumina layers at the bottom of the disk were chemically removed. A thin Au layer was then sputtered onto the open backside of the membrane to serve as an electrode for the subsequent Co electroplating. Cobalt NWs were grown at room temperature in an aqueous solution of 250 g/l CoSO_4_ and 40 g/l H_3_BO_3_. An Ag/AgCl reference electrode was combined into a three-electrode system in which a platinum electrode served as a counter electrode to conduct potentiostatic direct current electrodeposition. Electroplating was performed at −1 V. The pH of the solution was maintained at 3.5. The electroplating time was tuned such that the average length of the nanowires was approximately 10 μm.

### Structural characterization

An X′Pert PRO X-ray diffractometer was employed for the characterization of the crystal structure array of NWs. We performed θ–2θ scans with a scattering vector parallel to NW axes (perpendicular to the plane of AAO membrane). Prior to the XRD measurements, the Au metallic contact layer was partially removed using ion milling.

As prepared membranes were broken, sharp cross-sections were used for characterization by SEM. Planar sections of arrays for tomography and holography studies were prepared by the FIB protocol (see Supplementary Fig. S2).

Electron microscopy studies were performed on a TEM Titan G2 60–300 (FEI, Netherlands) operated at 300 kV. To study the crystal structure of individual NWs, the AAO membranes were dissolved in a Cr_2_O_3_/H_3_PO_4_-H_2_O solution at 40 °C and the Co NWs were dispersed in ethanol.

### Magnetic characterization

The magnetic properties of the nanowire arrays were studied using a vibrating sample magnetometer (EV7 KLA-Tencor). The magnetization curves were measured under magnetic fields up to 17 kOe with the field applied parallel (||) and perpendicular (⊥) to the NW axis.

LorTEM images and holograms of the remanent magnetic states of the planar sections of arrays were acquired using the Lorentz mode of the microscope. The Lorentz mode allows the specimen to be imaged in a field-free environment with the main objective lens of the microscope turned off. Off-axis electron holograms were acquired with an electron biprism operated typically near +200 V. Phase-shift reconstruction was done using a reference image. For the construction of magnetic induction maps, the cosine of the magnetic contribution to the phase shift was amplified to produce magnetic phase contours. Colors were added to show the direction of the magnetization rotation. The in-plane component of the magnetic induction was calculated from the holograms (see Supplementary Information for details).

MFM images were recorded in lift-off mode (100-nm distance) with an Agilent 5400 scanning probe microscope using standard atomic force microscopy nanosensor probes with a magnetic coating. A drop of ethanol containing Co NWs was placed on a clean Si wafer and then dried. A single NW was selected using SEM and its position was marked by FIB setup. MFM measurements were done at the remanent state after saturation in a 12-kOe magnetic field parallel to the NW axis.

### Simulations

The minimum-energy magnetic states of single-crystal, cylindrical Co NWs with 45- and 75-nm diameters and lengths between 20 and 1000 nm were simulated using the OOMMF package^32^; cell size was measured at 2 nm. The relative c-axis orientation with respect to the NW axis was chosen in plane with NW diameter. The initial magnetization was varied from being in plane with the diameter of NWs to being in parallel with the NW axis; in addition, NWs were in a state of random spin orientation. The ground state of the array presented in Figs 3 and 4 are also simulations. Shapes for the simulations were extracted from the tomography data shown in Fig. 7a. For the initial state, a random orientation of the spins in each cell was chosen.

The demagnetization process of NWs was simulated by the MagPar package with finite element discretization^31^. The average finite element discretization size was chosen to be 2 nm. The direction of the magnetocrystalline anisotropy was considered to be in agreement with TEM data (at 88° with respect to the NW axis). The simulated MFM image was evaluated from micromagnetic configurations as the divergence of the magnetization vector, which normally produces results that are indistinguishable from the evaluation of the magnetic force derivative.

LorTEM image simulations were performed by MALTS software^33^ using the following parameters: an accelerating voltage of 300 kV, a mean inner potential of *hcp* Co of −29.6 V, and defocus values of −200 and +200 μm, which corresponded with the parameters of our LorTEM experiments. An induction map similar to the one from the electron hologram was simulated using an LLG micromagnetic simulator^34^.

## Additional Information

**How to cite this article**: Ivanov, Y. P. *et al.* Single crystalline cylindrical nanowires – toward dense 3D arrays of magnetic vortices. *Sci. Rep.* **6**, 23844; doi: 10.1038/srep23844 (2016).

## Supplementary Material

Supplementary Information

Supplementary video 1

Supplementary video 2
