# Supplementary material for: Single crystalline cylindrical nanowires – toward dense 3D arrays of magnetic vortices
Source: Sci Rep. 2016 Mar 31;6:23844. doi: 10.1038/srep23844 (PMC4814901; doi:10.1038/srep23844)
Supplement: Supplementary Information [file srep23844-s1.doc]

Supplementary Information

**Single crystalline cylindrical nanowires – toward dense 3D arrays of magnetic vortices.**

Yurii P. Ivanov^1,2,*^, Andrey Chuvilin^3,4^, Laura G. Vivas^2,5^, Jurgen Kosel^1^, Oksana Chubykalo-Fesenko^2^, and Manuel Vázquez^2^

^1^King Abdullah University of Science and Technology, Thuwal, 23955, Saudi Arabia

^2^Instituto de Ciencia de Materiales de Madrid, CSIC, Cantoblanco, 28049, Madrid, Spain

^3^CIC nanoGUNE Consolider, Av. de Tolosa 76, 20018, San Sebastian, IKERBASQUE, ^4^Basque Foundation for Science, Maria Diaz de Haro 3, 48013, Bilbao, Spain

^5^Physics and Materials Science Research Unit, University of Luxembourg, 162A Avenue de la Faïencerie, L-1511, Luxembourg

*E-mail: [ivanov.yup@gmail.com](mailto:ivanov.yup@gmail.com)

**Cross-section of NW arrays.**


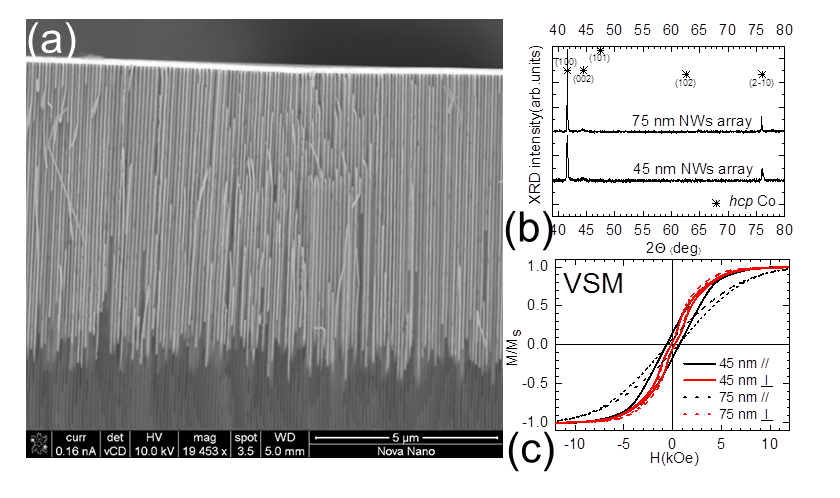


**Figure S1.** (a) Cross-sectional SEM image of Co (45 nm diam.) NWs embedded into an AAO membrane, (b) XRD and (c) VSM hysteresis loop for NW arrays with 45-nm and 75-nm diameters.

**Planar section of NW arrays.**

The AAO membranes with NWs embedded were broken and the resulting sharp cross-sections were used for the preparation of planar sections of arrays for tomo- and holographic studies by the FIB protocol, as shown in Figure S2.


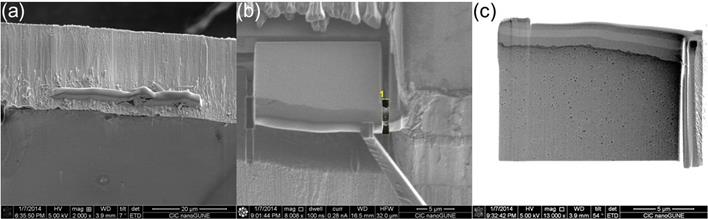


**Figure S2.** SEM images of the different stages of sample preparation for LorTEM and holography studies. (a) A layer of Pt is deposited on the AAO cross-section where the NWs are embedded, (b) the top view of the sample extracted from the AAO and (c) the final sample after FIB milling.

**CBED study.**

In addition to confirming monocrystallinity, CBED also monitors the orientation of the crystal structure with high precision along the NW axis. Figure S3 shows the TEM image of several NWs dispersed on the copper grid and the results of the CBED analysis (see also Supplementary Movies). The orientation of the crystal is reflected by the position of the Kikuchi lines with respect to the direction of the primary electron beam (marked by the red circle). Schematics on the image plots the projection of the beam direction on the Kikuchi map and its shift once the beam moves along the wire. As observed, the crystal rotates only slightly (~ 5 degrees) along several micrometers of the NW, proving the single-crystal nature of the NWs. Bending caused by preparing NWs for TEM studies may have caused the small tilts we observed.


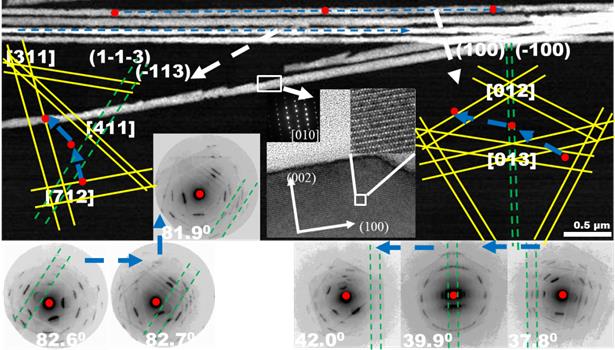


**Figure S3.** STEM images of a 75-nm diameter single-crystal Co NW and the CBED diffraction patterns taken at different positions along the single NW (scanning direction shown by blue arrows; the red spot represents the position of the central beam). The scheme shows the Kikuchi maps corresponding to each scanning line. The angles show relative orientation of the central beam with respect to the c-axis direction. The insert shows the HRTEM image and the corresponding SAED.

**Supplementary Movies 1, 2** are the series of CBED patterns obtained at different positions along an individual nanowire. The position of the Kikuchi lines does not change significantly as the probe is moved along the nanowires, indicating that the crystal orientation does not change significantly along NW length.

**Details of the in-plane component of the magnetic induction calculation.**

In general, the recorded phase shift was sensitive to both the in-plane component of the magnetic induction and to the electrostatic potential in the specimen. The height of the step in the magnetic contribution to the phase shift across NW diameter is given by the expression [1]


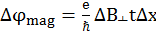
 (1)


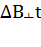
 is the projected component of the magnetic induction in the x direction, in-plane with NW diameter;
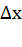
 is the distance where in-plane induction is changed on
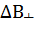
; and
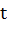
 is the length of the NW.

The electrostatic contribution is directly proportional to the thickness according to


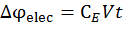
 (2)


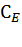
 is the interaction constant, which only depends on the electron energy (for 300 kV electrons,
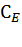
=6.526x10^-3^ rad/V nm) and
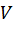
 is the inner potentials for the *hcp* Co (
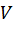
=29.6 V). If the thickness and the material of the specimen are homogenous, the electrostatic contribution should be constant inside the NW and any changes in the phase can be attributed to the magnetic field.

In the case of magnetic vortex state,
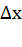
 in eq.1 is the radius of the NW (the in-plane component of induction is at a maximum at the edge of the NW and at zero in the center).

REFERENCE

[1] Kasama, T.; Dunin-Borkowski, R. E; Beleggia, M. *Electron Holography of Magnetic Materials, Holography - Different Fields of Application* Ch. 3 (InTech, 2011) DOI: 10.5772/22366.
